# Effect of Ankle Torque on the Ankle–Foot Orthosis Joint Design Sustainability

**DOI:** 10.3390/ma14112975

**Published:** 2021-05-31

**Authors:** Pruthvi Serrao, Vivek Kumar Dhimole, Chongdu Cho

**Affiliations:** Department of Mechanical Engineering, Inha University, 100 Inha-ro, Michuhol-gu, Incheon 22212, Korea; serrao@inha.edu (P.S.); vivek.dhimole@inha.edu (V.K.D.)

**Keywords:** ankle–foot orthosis (AFO), ankle joint torque, finite element analysis (FEA), mechanical design, worm gear

## Abstract

The ankle joint of a powered ankle–foot orthosis (PAFO) is a prominent component, as it must withstand the dynamic loading conditions during its service time, while delivering all the functional requirements such as reducing the metabolic effort during walking, minimizing the stress on the user’s joint, and improving the gait stability of the impaired subjects. More often, the life of an AFO is limited by the performance of its joint; hence, a careful design consideration and material selection are required to increase the AFO’s service life. In the present work, a compact AFO joint was designed based on a worm gear mechanism with steel and brass counterparts due to the fact of its large torque transfer capability in a single stage, enabling a compact joint. Further, it provided an added advantage of self-locking due to the large friction that prevents backdrive, which is beneficial for drop-foot recovery. The design was verified using nonlinear finite element analysis for maximum torque situations at the ankle joint during normal walking. The results indicate stress levels within its design performance; however, it is recommended to select high-grade structural steel for the ankle shaft as the highest stresses in AFO were located on it.

## 1. Introduction

A great deal of work is in progress on wearable orthotics, yet lightweight, portable, and efficiently designed products are not available on the commercial market. Hence, lightweight wearable orthotics for assistive motor functions have gained considerable popularity and research interest in recent years, which is quite evident from the numerous literature [1,2,3]. Ankle–foot orthosis is one of the highly sought areas of human augmentation and/or assistive flexion. Flexion of the foot about the ankle joint during walking is primarily used to define the biomechanics of walking; wherein the upward and downward movements of the foot about the ankle joint are termed dorsiflexion (DF) and plantarflexion (PF), respectively. Apart from flexion, the ankle joint also allows inward/outward turning of the sole, as well as internal/external rotation of the foot which are required for enhanced stability. The motion of the ankle occurs primarily in the sagittal plane, with both PF and DF occurring predominantly at the tibiotalar joint. Although the range of these angles varies from person to person, it must be precisely measured during an individual’s gait, as the same values will be controlled by an externally powered flexion mechanism during assisted walking [4]. Ankle flexion assist is usually sought for (a) extending the active years of motor functionality in the elderly population [5], (b) reducing the metabolic cost of walking by providing assisted walking in the case of a healthy individual [6], and (c) improving the motor functions of persons suffering from impaired gait capabilities [7,8,9]. All of the above functionalities are expected but with a compact setup, featuring a portable design and an energy-efficient powering mechanism.

Numerous types of powering mechanism have been employed for the plantar flexion such as electric actuators in a series with a spring element (SEA: series elastic actuator) that uses the motors for powering flexion [10,11]; pneumatic actuators (PAM: pneumatic artificial muscle) that use compressed air for actuation [7,12]; hydraulic actuators that use hydraulic pressure generated through pumps for actuation [13]. Most of these orthoses are developed with an auxiliary powering unit and are limited to research or rehabilitation PAFO applications [8]. Thus, the major drawback of the existing PAFOs is their excessive weight; although intended to enhance walking efficiency, they instead end up causing discomfort or fatigue for individuals when worn for a long duration due to the excessive weight. In addition, a high torque requirement for efficient flexion powering in a compact setup makes it difficult to achieve the same in a portable design [1]. Mass added at the lower limbs increases the moment of inertia of the legs, which increments the metabolic cost of walking. It is estimated that an added weight of 2 kg to a human foot can increase the oxygen consumption rate by up to 30%, which would result in increased metabolic cost; thus, it is recommended to limit the weight at the ankle to under 1.2 kg [14].

In the present work, a compact worm gear mechanism-based ankle flexion powering design is proposed. The worm gear was used due to the fact of its ability for large torque multiplication in a single stage [15] along with its self-locking ability. The self-locking depends on the coefficient of friction between the mating gears as well as the lead angle of the worm screw. The movement between the worm screw and the wheel gear faces can be expected to occur by entirely sliding without any rolling and, hence, the resulting high friction will not allow backdrive [16]. Large frictions usually tend to reduce the service life of the gears at relatively high speeds; however, since the ankle flexion speed is considerably low, it is not of concern. Further, if the self-locking feature is not the intended functionality, then multi-start worm screws can be used for faster actuation at the expense of torque; the multi-start threads have a steeper helix angle, thus offering less friction and are less likely to be self-locking.

The contents of the article are structured in the following order: (a) the design of the orthosis, (b) the methodology implemented for design verification, and (c) the test results. The Section 2 describes the design of the AFO, whereas Section 3 enumerates the methodology implemented for evaluating the design. The design feature includes a compact flexion powering mechanism and a lightweight wearable orthosis structure along with the design considerations. Further, specific design features and material selection were considered to minimize the effect of stresses on the structure while maintaining the overall weight of the structure to a minimum prescribed for an efficient ankle assist. Although suitable for achieving the required compact setup, the proposed mechanism needed to be analyzed for its functional performance and design sustainability. Hence, based on the maximum stress conditions encountered due to the kinetic forces about the ankle joint during normal walking, the mechanism was subjected to nonlinear numerical analysis to evaluate the effect of stresses on the AFO structural components. A short look at future design considerations and modifications based on the findings from the present research are also highlighted in the results and conclusions.

## 2. Design of a Joint for Powered Ankle–Foot Orthosis

The design of the AFO structure including the flexion mechanism was performed using SOLIDWORKS 3D modeling software package. The overall system weight of the orthosis, including the motor, was measured at approximately 1.1 kg, which is less than the prescribed weight limit for an effective assistive PAFO.

The important factors to consider for the design of an ankle orthosis joint are:

1. Compact ankle flexion powering mechanism;
2. Structural design to minimize and/or withstand the ankle torque;
3. Material selection for optimum strength to the overall orthosis weight.

### 2.1. Flexion Powering Mechanism

**Figure 1:**
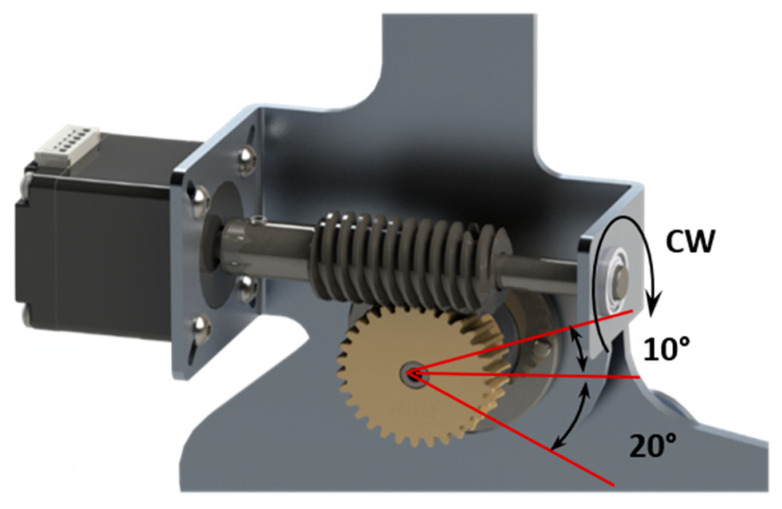
Ankle flexion mechanism.

An ankle joint complex with a worm gear mechanism in a speed reducing configuration, as shown in Figure 1, was used for powering the ankle flexion; wherein the gear mechanism is driven by a compact motor. The mechanism offer the two-fold advantages of (i) a large torque transmission, which implies that a small-capacity motor can drive the mechanism, and (ii) self-locking ability, which can be beneficial as a solution to the drop-foot condition [10,17]. Self-locking is when the worm wheel cannot drive the worm screw, which is also referred to as backdrive. The gear reduction ratio, the size of gears, and the motor were selected based on the ankle kinematic parameters as show in Table 1.

### 2.2. Orthosis Structure and Joint Design

The AFO structure, comprising an upright frame and a foot frame, was fabricated using a sufficiently thick sheet metal design. Being positioned parallel to the sagittal plane, the orthosis frame provides the stiffness needed for powering ankle flexion while retaining the flexibility about the joint for moderate out-of-plane movements. In addition, the flexible upright frame design, which allows a certain degree of elastic deflection, helps to minimize the stress on the ankle joint of both the user and the orthosis, in particular, and the overall AFO structure, in general.

**Figure 2:**
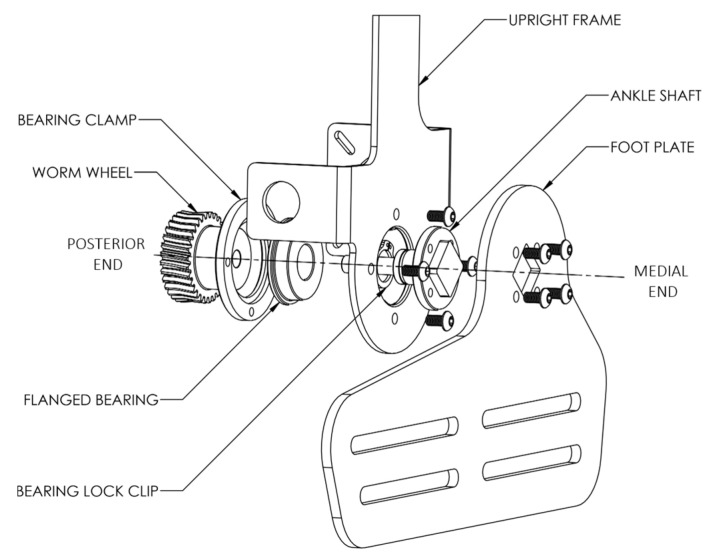
Ankle joint complex for the right foot (exploded view).

The AFO joint was constructed about a stainless-steel shaft that was firmly locked onto the square hole of the foot frame and fastened to it firmly on the medial end; while the shaft running through a flanged bearing mounted on the upright frame, it held the worm wheel on the posterior end. The upright frame was a fixed member of the AFO being strapped to the leg, whereas the foot frame moved about the ankle joint and was firmly strapped to the foot. The motor drove the worm wheel through a worm screw, powering the ankle flexion during walking. An exploded view detailing the internal components of the ankle joint and the structure of the orthosis is shown in Figure 2.

### 2.3. Material Selection

Proper material selection to meet the specific functional requirement is very crucial for an optimum design performance. As the frame constitutes most of the AFO’s gross weight, a structural-grade aluminum alloy was selected to a deliver high strength-to-weight ratio; whereas the components at the ankle joint, such as the joint shaft and linkages, being subjected to considerably higher stress levels, were made of ASTM A36 structural steel. Regarding the worm gears, it is customary to select a hard material, mostly surface-hardened steel, for the worm screw and a softer material, such as brass/bronze, for its mating counterpart to minimize the friction and wear on the worm tooth. The materials selected for the orthosis and their properties considered for the analysis are listed in Table 2.

## 3. Analysis of the Ankle–Foot Orthosis Joint Stress

A mechanical structure must always be thoroughly tested for its design feasibility and worthiness and, hence, the developed 3D model was exported to the ANSYS software package to perform an FE analysis. The AFO joint in the present analysis was tested for its durability against the torque exerted by a user about the ankle joint during normal walking. First, the applied torque was theoretically estimated with added reference to the literature, and then the stress analysis of the AFO ankle joint was performed for the calculated torque values to evaluate its design stability and performance characteristics.

### 3.1. Ankle Joint Torque Estimation

The AFO experiences the ground reaction force (GRF) during walking, which is the force exerted by the ground on the foot as it makes contact with it due to the gravitational attraction. While walking, the GRF provides a measure of the impact that a body experiences when the foot makes contacts with the ground. For a static condition, the GRF corresponds to the person’s weight, but while in motion, the GRF increases due to the acceleration of the body. For normal walking, the GRF is the sum of the person’s weight and the acceleration of the center of mass (CM) as shown in Equation (1) [19].

**Figure 3:**
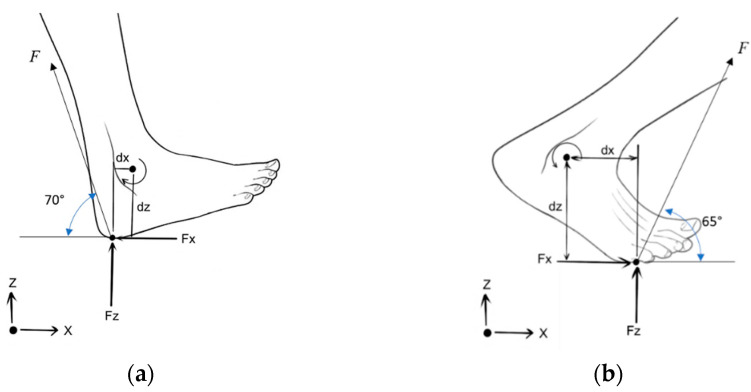
Force components and CoP during: (**a**) heel-strike; (**b**) toe-off.

(1) GRF=M(g+a),

where *M* is a person’s mass, *g* is the acceleration due to the fact of gravity, and *a* is the vertical acceleration of the CM. The GRF for a person weighing 80 kg while considering an average walking acceleration of 0.7 m/s^2^ will be 80 (9.81 + 0.7) = 840 N. However, the entire force may not be applied on the foot due to the weight being distributed among both legs and considering the inertia depending on the speed of walking. Thus, considering 80% of the total force applied on the foot, we obtain a force of 672 N, for which the horizontal and vertical components of the forces can be calculated with respect to the foot reference planes. By knowing the force components acting about the sagittal plane at the center of pressure (CoP) during a specific phase of walking and the distance of the CoP from the ankle joint, as shown in Figure 3, we can calculate the torque generated about the ankle joint [20]. The resulting torque can be measured by Equation (2).

(2) $$\mathrm{Torque}\;(\mathrm{Nm}),\;T=F_{x}dz+F_{z}dx,$$

where *F_x_* and *F_z_* are components of the force along the *x-* and *z*-directions, respectively. Whereas *dx* and *dz* are the distance of the CoPs from the ankle joint measured along *x-* and *z*-axes, respectively.

The applied load or torque about the ankle joint has a variable nature that can be attributed to the variance in the type of foot contact with the ground at ankle angles corresponding to the different phases of the walking gait. Accordingly, heel-strike and toe-off, as indicated in Table 3, are the two prominent phases that generate maximum torque about the ankle joint. Hence, these two phases will be considered to evaluate the effect of torque on the structural integrity of the AFO joint. For these two conditions, the applied torque can be calculated for an average user based on his weight and anthropometric data as shown in Table 4. In addition, similar findings are observed in the literature [4,21] for the applied torque about the ankle joint during heel-strike and toe-off phases of a gait during normal walking conditions.

### 3.2. Stress Analysis of the AFO Joint

Nonlinear analysis was considered for the AFO ankle joint using the Newton–Raphson method, and the mesh model was created using pure penalty contact formulation while considering the coefficient of friction between the mating gears.

#### 3.2.1. Material Model

**Figure 4:**
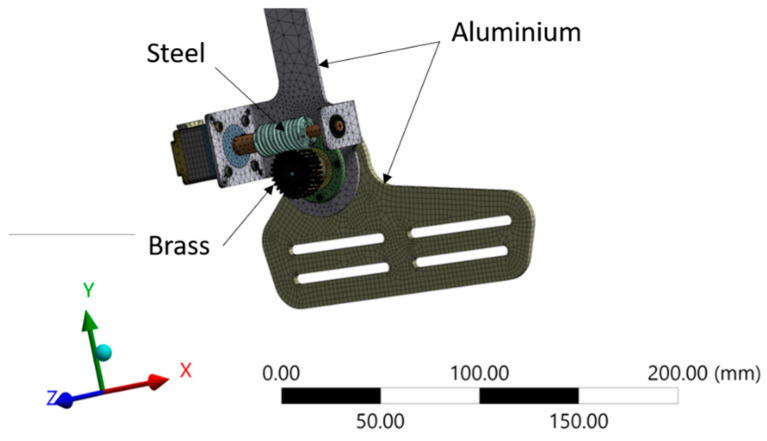
Mesh model of the AFO ankle joint.

For both cases, the material model, as shown in Figure 4, with a multizone modeling strategy was applied because of the complexity of the model. Altogether, the model contains hexahedral as well as tetrahedral elements; some thin parts were meshed with SHELL181 mesh including part thickness. The SOLID187 element type was selected for tetrahedral meshing, which is a higher-order 3D, 10-node element; whereas for hexahedral meshing, SOLID186 element, which is a higher-order 3D, 20-node element, was selected; both SOLID187 and SOLID186 have quadratic displacement behavior and are well suited to modeling irregular meshes. The mesh was completed with 4,845,801 nodes and 3,186,059 elements. CONTA174 and TARGE170 were used as contact elements, where TARGE 170 was used to represent various 3D “target” surfaces for the CONTA 174, and CONTA172 was used to define contact and sliding between 3D target surfaces and a deformable surface. The contact elements themselves overlayed the solid and shell elements and, thus, defined the boundary of a deformable body and were potentially in contact with the target surface, defined by TARGE170 [23]. 

#### 3.2.2. Contact Formulation

To ensure contact compatibility so that the contacting bodies, such as gears at the ankle joint, did not interpenetrate, a pure penalty contact formulation, as shown in Equation (3), was used [24].

(3) $$F_{n}=k_{n}\cdot x_{p},$$

where *F_n_* is the contact force, *k_n_* is the contact stiffness, and *x_p_* is the penetration depth. To ensure accurate results, the penetration depth should be as small as possible, which depends on the magnitude of the contact stiffness.

Moreover, a frictional contact formulation was defined with 0.2 as the coefficient of friction, μ for the mating worm and worm wheel set. The value was selected for a maximum friction case; however, the coefficient of friction of a material is difficult to evaluate and depends on various factors such as sliding speed, gear specifications (lead angle, module, etc.), and the lubrication conditions. Normally, between a steel worm screw and a brass/bronze worm wheel, the value of μ is approximately 0.15 [25]. Further, given the very slow ankle flexion speeds during walking, the stress due to the friction between the worm gears may be expected to be considerably low or negligible.

#### 3.2.3. Formulation of Solution for Nonlinear FEA Equation

The analysis was conducted based on the Newton–Raphson (N–R) method as a solution to the nonlinear finite element equation. The N–R iteration is commonly used for nonlinear static structural analysis of a multiple degrees of freedom system.

**Figure 5:**
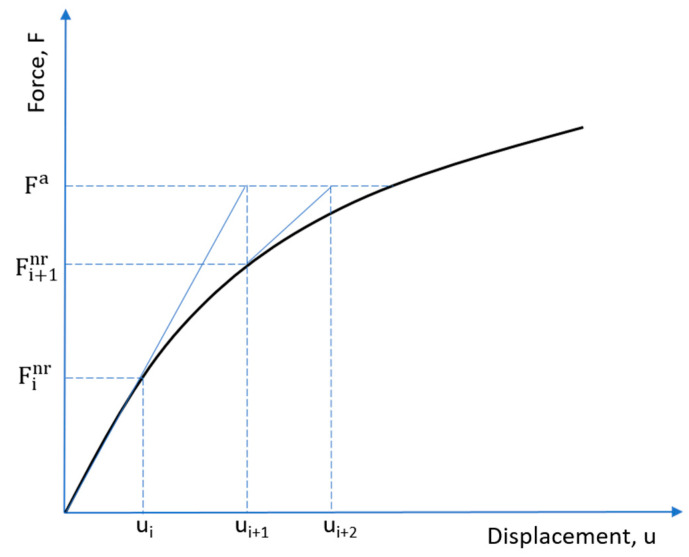
Newton–Raphson iteration [26].

The governing equation for a finite element analysis may be written as:

(4) $$\left[K\right]\left\{U\right\}=\left\{F_{a}\right\},$$

where, [*K*] is the stiffness matrix, {*U*} is the displacement vector of unknown degrees of freedom, and {*F_a_*} is the applied load. If the stiffness matrix itself is a function of the displacement vector, then the Equation (4) is termed a nonlinear equation; then, N–R iteration applied to obtain the root of the nonlinear equation may be written as:

(5) $$\left[K_{i}^{T}\right]\left\{\Delta u_{i}\right\}=\left\{F_{a}\right\}-\left\{F_{i}^{nr}\right\},$$

(6) $$\left\{u_{i+1}\right\}=\left\{u_{i}\right\}+\left\{\Delta u_{i}\right\},$$

where [*K_i_^T^*] is the tangent stiffness matrix, *i* represents the current iteration; {*u_i_*} is the displacement vector; {*F_i_^nr^*} is the restoring force vector calculated from element stresses. In the iteration process, {Δ*u_i_*} is calculated from Equation (5) with the known element stiffness matrix and element stresses; next, the calculated {Δ*u_i_*} is added to {*u_i_*} to get the next approximation {*u_i+_*_1_}. This process is repeated till we obtain the convergence as shown in Figure 5 [26].

## 4. Results and Discussion

The ANSYS software package was used for performing the FE analysis and simulation. The complete analysis of the AFO joint model took approximately 5 h for each case. The applied boundary conditions and the results of the static structural analysis for both the heel-strike and toe-off phases are presented below. 

### 4.1. Static Structural Analysis during Heel-Strike

**Figure 6:**
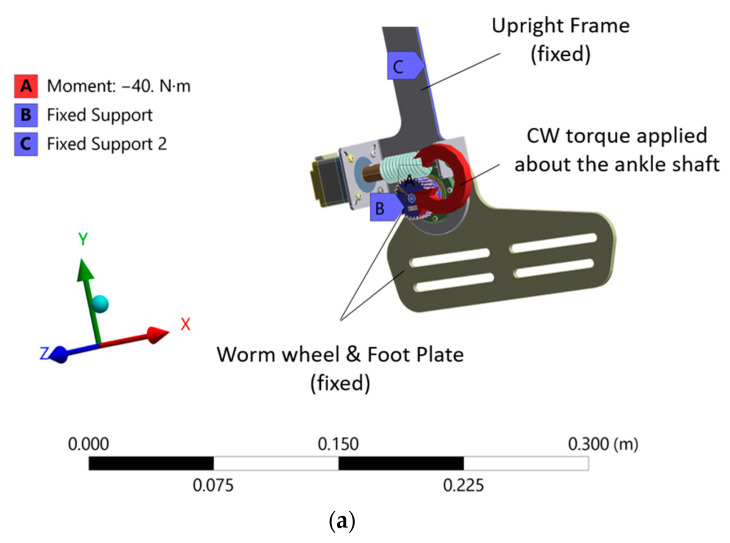

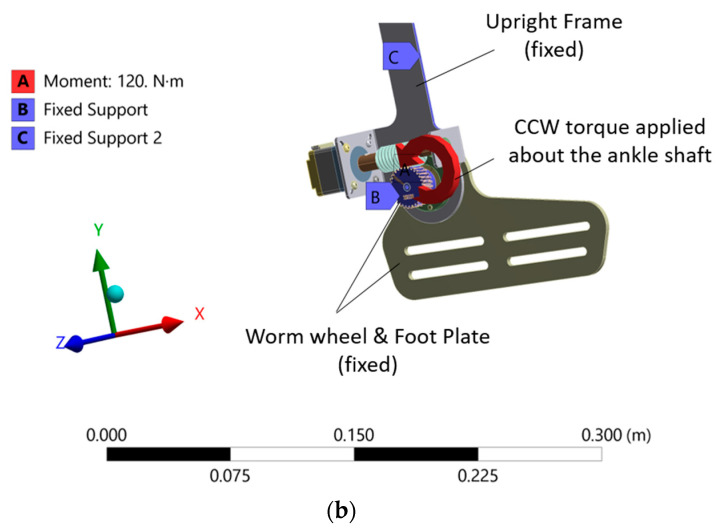
AFO joint with load and boundary conditions: (**a**) heel-strike phase; (**b**) toe-off phase.

A torque of approximately 40 Nm expected to be generated about a human ankle joint, as calculated previously during the heel-strike phase, was taken as the applied torque input for the AFO structure. The torque was acting in the clockwise direction due to the PF torque about the ankle joint. By keeping the remaining structure fixed against rotation, as shown in Figure 6a, the entire structure was evaluated for the equivalent Von-Mises stress generated about the ankle joint. The specified fixed support condition arises due to the restricted backdrive of the worm gear, wherein any torque applied by the human ankle prevents the rotation of the worm wheel and, hence, the applied torque results in the structure being subjected to the corresponding stress values. It is to be noted that, with the PF activated by the motor, the stress experienced by the structure will be drastically reduced.

**Figure 7:**
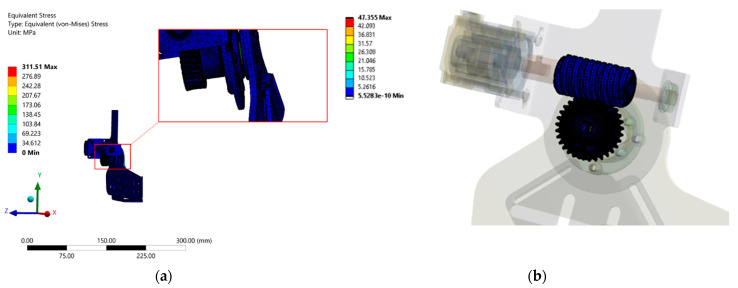
Equivalent Von-Mises stress for the heel-strike phase: (**a**) stress on the entire structure with an enlarged view; (**b**) stress at the gear contact zone.

The results, as seen in Figure 7a, showed a maximum average stress of approximately 75 MPa, which is seen in its enlarged view about the ankle shaft connecting the worm and, hence, is of interest for the design consideration. Overall, the stress level on all of the parts was considerably low, indicating design feasibility. Additionally, a maximum average stress of 17 MPa was observed at the gear contact despite the maximum frictional condition between the mating gears. The stress at the gear contact can be seen in Figure 7b.

### 4.2. Static Structural Analysis during Toe-Off

For a toe-off phase, as shown in Figure 6b, an applied torque of 120 Nm, as measured from Section 3.1 previously, was taken as the input at the AFO ankle joint, which created a counterclockwise moment due to the DF torque. The fixed support condition similar to the heel-strike phase was maintained here as well due to the restricted backdrive; however, the input torque during a toe-off phase is comparatively larger. With powered flexion in action during toe-off, the stress on the structure will be significantly less. The applied load on the orthosis during both heel-strike and toe-off phases are only considered to simulate the static condition.

**Figure 8:**
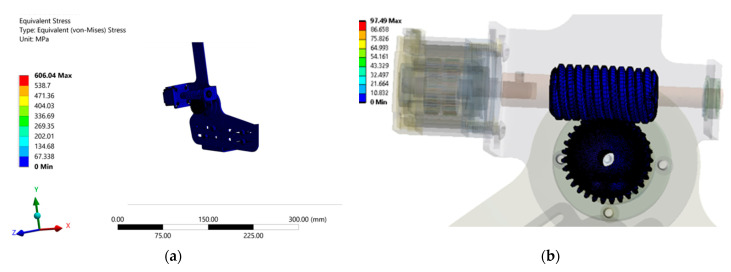
Equivalent Von-Mises stress for the toe-off phase: (**a**) stress on the entire structure; (**b**) stress at the gear contact zone.

Even during the toe-off phase, a similar scenario was observed with a maximum average stress of approximately 130 MPa at the ankle joint shaft as shown in Figure 8a, whereas the average stress at the gear contact was approximately 30 MPa from Figure 8b. All the components of the orthosis were subjected to elastic deformation only for the applied load, and the stress level was much lower in all the parts, with the ankle shaft being the component of interest for design consideration. The ankle joint poses a high degree of complexity due to the fact of its geometrical features; however, in reality, with sufficient dimensional tolerance, consideration of fillets and chamfer for minimized stress concentration, and adequate connections, fasteners and joining techniques, etc., the overall stress on the parts can be considerably reduced.

### 4.3. Design Outcome and Future Considerations

The analysis results obtained for the orthosis are useful for validating the preliminary design of the ankle joint, understanding the suitability of material selection for the components, reviewing the mechanism employed for ankle flexion, and identifying the crucial components for a possible design modification.

For both cases, it was observed that the maximum stress region was confined within the ankle joint shaft in an orthosis and, hence, due considerations will be made during the prototype design to minimize the stress concentration about the ankle shaft and to consider optimizing the shaft diameter based on the ankle joint kinetics. The current structure, however, minimizes the stress on the joints due to the fact of its flexible design, allowing for large elastic deformation, will include a serial spring element in the second prototype design for enhanced ankle flexibility; this is expected to further reduce the stress on the ankle shaft. The design and results from the present study will serve as a reference for those considering the worm gear mechanism as a means for powering the ankle flexion. 

Further studies based on fatigue analysis as a second phase verification on the orthosis and the ankle shaft, in particular, will be considered, as the components are subjected to cyclic stresses during walking. Although moderate stress levels are induced on the ankle joint, the interaction of multiple components at the joint may diminish the fatigue life of the ankle shaft; the minimum suitable diameter and the material selection and processing considerations will be included for the study. 

With satisfactory test results, a test model is being developed based on the current design that uses a surface-hardened worm screw, ground and mated with a relatively soft worm wheel made of phosphorous bronze; thus, much lower stress levels may be expected for the actual test setup at the mating gear part. The ankle shaft will be made of high-grade structural steel to withstand high stress and fatigue loading. The developed orthosis will be tested for its assistive ability in enhancing/regaining the motor functions intended to help the elderly or persons suffering from diminished ankle flexion performance. 

## 5. Conclusions

A simple, yet efficient flexion powering joint design was suggested in the present work for an AFO. The proposed design for powering the plantar flexion was based on a worm gear mechanism, which is advantageous due to the fact of its large torque transmission capability with self-locking capability. Moreover, the design was subjected to a nonlinear finite element analysis based on the maximum torque generated during the heel-strike and toe-off phases of a gait during normal walking; the ankle torques generated during normal walking were calculated to be 40 Nm CW and 120 Nm CCW during the heel-strike and toe-off phases, respectively. The structures were subjected to safe stress levels indicating a structurally feasible design, wherein the maximum stress values of 75 MPa and 130 MPa were observed at the ankle joint shaft during nonlinear FEA analysis for the heel-strike and toe-off phases, respectively. The orthosis design was structurally verified, and a test prototype based on the current flexion powering design will be fabricated and tested, a high-grade structural steel is recommended for the ankle joint shaft, with carburized and ground steel worm screw, mating with a phosphorous bronze worm wheel for better service life expectancy from an AFO. Structural design modifications of the orthosis are considered for the future work by including a serial spring element to further minimize the stress on the joint and improve the stability. 

## Figures and Tables

**Table 1:** Worm gear and joint kinematic parameters.

Parameters	Values
Reduction ratio	1:15
Worm rotation per unit degree ankle tilt	0.042
Ankle angular velocity ^1^, rad/s	1.25
Average motor speed ^2^ for normal walking at 0.7 m/s, rps	3

^1^ Calculated as the product of total gait angle (degrees) and gait time with a multiplication factor of π/180 for converting degrees to radians. The experimentally measured average ankle movement angle during one gait cycle was approximately 123 degrees for a gait time of 1.7 s. ^2^ Calculated as the product of ankle angular velocity (rad/s) and the reduction ratio with a multiplication factor of 60/2π for converting rad/s to rpm.

**Table 2:** Ankle joint complex materials and their mechanical properties [18].

Material Properties	Unit	Structure	Worm Wheel	Worm Screw
		(A6061)	(Brass)	(A36 Steel)
Yield Strength	MPa	280	88	250
Tensile Strength	MPa	310	278	420
Young’s Modulus	GPa	71	96	200
Poisson’s Ratio	-	0.33	0.345	0.3

**Table 3:** Ankle torque measurement [22].

Parameters	Heel-Strike	Mid-Stance	Toe-Off
GRF	behind ankle	through ankle	in front of ankle
Torque	causes PF ^1^ torque	no torque	causes DF ^2^ torque

^1^ Plantarflexion (PF) and ^2^ dorsiflexion (DF) refer to the downward and upward movements of the foot about the ankle joint, respectively.

**Table 4:** Ankle torque calculation.

Parameters	Heel-Strike	Toe-Off
Force angle	70°	65°
Force components ^1^	*F_X_ = F* cos*θ* = 230 N	*F_X_ = F* cos*θ* = 284 N
	*F_Z_ = F* sin*θ* = 632 N	*F_Z_ = F* sin*θ* = 609 N
CoP distance with respect to the ankle joint	*dx* = 0.02 m	*dx* = 0.12 m
	*dz* = 0.12 m	*dz* = 0.16 m
Torque about ankle, Nm	(−*F_X_*·*dz*) + (−*F_Z_*·*dx*) = −40	(*F_X_*·*dz*) + (*F_Z_*·*dx*) = 120
Orientation	Clockwise (CW)	Counterclockwise (CCW)

^1^ The force components were calculated based on the applied force of 672 N, previously calculated using Equation (1).

## Data Availability

Not applicable.

## References

[1] Moltedo M., Baček T., Verstraten T., Rodriguez-Guerrero C., Vanderborght B., Lefeber D. (2018). Powered ankle-foot orthoses: The effects of the assistance on healthy and impaired users while walking. J. Neuroeng. Rehabil..

[2] Adiputra D., Nazmi N., Bahiuddin I., Ubaidillah U., Imaduddin F., Rahman M.A.A., Mazlan S.A., Zamzuri H. (2019). A review on the control of the mechanical properties of Ankle Foot Orthosis for gait assistance. Actuators.

[3] Frisoli A. (2004). Wearable robots. Technol. Rev..

[4] Brockett C.L., Chapman G.J. (2016). Biomechanics of the ankle. Orthop. Trauma.

[5] Healy A., Farmer S., Pandyan A., Chockalingam N. (2018). A systematic review of randomised controlled trials assessing effectiveness of prosthetic and orthotic interventions. PLoS ONE.

[6] Galle S., Malcolm P., Collins S.H., De Clercq D. (2017). Reducing the metabolic cost of walking with an ankle exoskeleton: Interaction between actuation timing and power. J. Neuroeng. Rehabil..

[7] Shorter K.A., Kogler G.F., Loth E., Durfee W.K., Hsiao-Wecksler E.T. (2011). A portable powered ankle-foot orthosis for rehabilitation. J. Rehabil. Res. Dev..

[8] Bae J., De Rossi S.M.M., O’Donnel K., Hendron K., Awad L.N., Teles Dos Santos T.R., De Araujo V.L., Ding Y., Holt K., Ellis T.D. (2015). Soft Exosuit For Poststroke Gait Assistance. IEEE Int. Conf. Rehabil. Robot..

[9] Yeung L.F., Ockenfeld C., Pang M.K., Wai H.W., Soo O.Y., Li S.W., Tong K.Y. (2017). Design of an exoskeleton ankle robot for robot-assisted gait training of stroke patients. IEEE Int. Conf. Rehabil. Robot..

[10] Blaya J.A., Herr H. (2004). Adaptive Control of a Variable-Impedance Ankle-Foot Orthosis to Assist Drop-Foot Gait. IEEE Trans. Neural Syst. Rehabil. Eng..

[11] Yeung L.F., Ockenfeld C., Pang M.K., Wai H.W., Soo O.Y., Li S.W., Tong K.Y. (2018). Randomized controlled trial of robot-assisted gait training with dorsiflexion assistance on chronic stroke patients wearing ankle-foot-orthosis. J. Neuroeng. Rehabil..

[12] Takahashi K.Z., Lewek M.D., Sawicki G.S. (2015). A neuromechanics-based powered ankle exoskeleton to assist walking post-stroke: A feasibility study. J. Neuroeng. Rehabil..

[13] Neubauer B.C., Nath J., Durfee W.K. Design of a portable hydraulic ankle-foot orthosis. Proceedings of the 2014 36th Annual International Conference of the IEEE Engineering in Medicine and Biology Society.

[14] Waters R.L., Mulroy S. (1999). The energy expenditure of normal and pathologic gait. Gait Posture.

[15] Magyar B., Sauer B. Calculation of the efficiency of worm gear drives. Proceedings of the International Gear Conference.

[16] Carvill J., Cullum R.D. (1994). Power Units and Transmission. Mechanical Engineer’s Reference Book.

[17] Alam M., Choudhury I.A., Mamat A. (2014). Bin Mechanism and design analysis of articulated ankle foot orthoses for drop-foot. Sci. World J..

[18] Material Property Data. http://www.matweb.com/.

[19] Blaya J.A. (2003). Force-Controllable Ankle Foot Orthosis (AFO) to Assist Drop Foot Gait. Master’s Thesis.

[20] Meadows B., Bowers R. (2019). Biomechanics of the Hip, Knee, and Ankle.

[21] Grimmer M., Seyfarth A. (2014). Mimicking Human-Like Leg Function in Prosthetic Limbs. Neuro-Robotics.

[22] Webster J.B., Darter B.J. (2019). Principles of Normal and Pathologic Gait.

[23] ANSYS Inc. (2010). ANSYS Mechanical APDL Modeling and Meshing Guide.

[24] Yoo D.H., Jang B.S., Yim K.H. (2017). Nonlinear finite element analysis of failure modes and ultimate strength of flexible pipes. Mar. Struct..

[25] Fontanari V., Benedetti M., Straffelini G., Girardi C., Giordanino L. (2013). Tribological behavior of the bronze-steel pair for worm gearing. Wear.

[26] ANSYS Inc. (1999). ANSYS Theory Reference—Release 5.6.

